# Retinal Vascularization Analysis on Optical Coherence Tomography Angiography before and after Intraretinal or Subretinal Fluid Resorption in Exudative Age-Related Macular Degeneration: A Pilot Study

**DOI:** 10.3390/jcm10071524

**Published:** 2021-04-06

**Authors:** Thibaud Mathis, Sarra Dimassi, Olivier Loria, Aditya Sudhalkar, Alper Bilgic, Philippe Denis, Pierre Pradat, Laurent Kodjikian

**Affiliations:** 1 Service d’Ophtalmologie, Centre Hospitalier Universitaire de la Croix-Rousse, Hospices Civils de Lyon, Université Claude Bernard Lyon 1, 69004 Lyon, France; thibaud.mathis@chu-lyon.fr (T.M.); dimassarra@hotmail.com (S.D.); olivier.loria@chu-lyon.fr (O.L.); philippe.denis@chu-lyon.fr (P.D.); 2 UMR-CNRS 5510, Matéis, Villeurbane, 69100 Lyon, France; 3 Alphavision Augenzentrum, 27568 Bremerhaven, Germany; adityasudhalkar@icloud.com (A.S.); drbilgicalper@yahoo.com (A.B.); 4 MS Sudhalkar Medical Research Foundation, Baroda 390001, India; 5 Centre de Recherche Clinique, Centre Hospitalier Universitaire de la Croix-Rousse, Hospices Civils de Lyon, Université Claude Bernard Lyon 1, 69004 Lyon, France; pierre.pradat@chu-lyon.fr

**Keywords:** age-related macular degeneration, exudation, optical coherence tomography angiography

## Abstract

The aim was to analyze the variations in macular vascularization on optical coherence tomography angiography (OCTA) according to the presence of intraretinal fluid (IRF) induced by exudative age-related macular degeneration (AMD). We included exudative AMD patients with IRF and/or subretinal fluid (SRF) and age-matched control eyes. All patients underwent a macular 6 × 6 mm swept-source OCTA. The mean perfusion density (MPD) and mean vascular density (MVD) were calculated in the superficial (SCP) and the deep (DCP) capillary plexus at two timepoints: during an episode of exudation (T0) and after its total resorption (T1). A total of 22 eyes in the IRF ± SRF group, 11 eyes in the SRF group and 11 eyes in the healthy group were analyzed. At T0, the IRF ± SRF group showed significantly lower MPD and MVD than healthy eyes in the SCP (*p* < 0.001) and DCP (*p* < 0.001). At T1, MPD and MVD significantly increased from T0 in the SCP (*p* = 0.027 and *p* = 0.0093) and DCP (*p* = 0.013 and *p* = 0.046) but remained statistically lower than in the healthy eyes. For the SRF group, only the DCP showed significantly lower MPD (*p* = 0.012) and MVD (*p* = 0.046) in comparison to the healthy eyes at T0. The present study shows that retinal vascular changes do occur in the case of exudative AMD.

## 1. Introduction

Age-related macular degeneration (AMD) is the leading cause of blindness worldwide [1]. Its pathophysiology is multifactorial and remains unclear [2]. Unlike the atrophic form of the disease, neovascular AMD benefits from treatment with intravitreal injections (IVIs) of anti-vascular endothelial growth factor (anti-VEGF). There are two types of exudation: the accumulation of subretinal fluid (SRF) associated with a breakdown of the outer blood–retinal barrier, and the accumulation of intraretinal fluid (IRF) characterized by the presence of intraretinal cysts. The latter is known to be associated with a worse functional prognosis [3,4,5]. Macular edema (ME) is a common manifestation of several retinal pathologies, its physiopathology is complex and involves the dysregulation of the blood–retinal barrier and hemodynamic phenomenon [6].

Optical coherence tomography angiography (OCTA) is a recent diagnosis tool in ophthalmology for the non-invasive examination of the retinal and choroidal microvasculature [7,8]. It has been shown on OCTA that retinal vascularization can change before and after IRF resorption in patients with retinal vascular disease. For example, in chronic diabetic ME (DME), cystoid spaces co-localized with capillary loss areas and no reperfusion occurred after DME resolution [9]. The same findings have been observed for retinal vein occlusion (RVO) [10]. However, unlike DME and RVO, macular vessel density after the resolution of an acute pseudophakic cystoid ME did not differ from the control eyes [11]. Several studies based on OCTA have analyzed the variations in the choroidal neovessels before and after anti-VEGF treatment [12,13,14]. However, little is known about changes in retinal vascularization in cases of IRF induced by neovascular AMD.

The aim of the present pilot study was to analyze the variations in retinal macular vascularization on OCTA according to the presence of fluid (IRF and/or SRF) induced by neovascular AMD, in comparison to age-matched control eyes. The secondary objectives included the change in retinal macular vascularization over time after fluid resorption.

## 2. Materials and Methods

### 2.1. Subjects and Follow-Up

An observational prospective case–control study was performed in our ophthalmology department at the Croix-Rousse University Hospital in Lyon, France, between January 2019 and December 2019. This study complied with the tenets of the Declaration of Helsinski and was approved on 15 January 2021, by an international review board (Ethics Committee of the French Society of Ophthalmology, IRB 00008855 Société Française d’Ophtalmologie IRB#1). Patients gave their informed consent to participate in the study.

Patients aged 65 years or older with a diagnosis of exudative AMD were included in the study. Best corrected visual acuity (BCVA) was measured using the Early Treatment Diabetic Retinopathy Study (ETDRS) chart. All patients underwent a complete ophthalmological examination including slit lamp and fundus examination, spectral domain– (SD–) OCT, fluorescein angiography (FA), indocyanine green angiography (ICGA) (Heidelberg Spectralis, Heidelberg Engineering, Heidelberg, Germany) and swept source– (SS–) OCTA (PLEX Elite 9000, Carl Zeiss Meditec, Dublin, CA, USA). All the images obtained were analyzed by two retinal specialist graders who confirmed the diagnosis of exudative AMD and determined the neovascular subtype, as described by Freund et al. [15]. The exclusion criteria were the presence of any other eye disease, including myopia ≥ 6D, or ophthalmic surgery except for cataract extraction. At the time of inclusion, eyes with retinal hemorrhage were not included due to possible artifact on OCTA. Patients with systemic high blood pressure, diabetes, smokers and patients with other systemic vascular diseases were not included.

Two subgroups of patients were included. The first group was composed only of eyes presenting significant IRF with at least three cystic spaces visualized on SD–OCT with or without SRF (group IRF ± SRF). The second group of patients with a diagnosis of exudative AMD and SRF without IRF (group SRF) was compared to the first group. Patients in the SRF group were matched for age and disease duration with patients in the IRF ± SRF group. A third control group was composed of age-matched (±5 years) healthy individuals with no ocular or systemic diseases, who were recruited and attended a single visit during which they underwent the ophthalmological exams including OCTA. For all patients included in the IRF and SRF groups, the OCTA images were analyzed during an episode of exudation with IRF and/or SRF (T0) and after its total resorption (T1). Any patients who developed another eye disease or underwent eye surgery between T0 and T1 were excluded/withdrawn from the study.

### 2.2. Data Collection

Demographic data were obtained from the medical charts on the day of inclusion. Data collected were age, sex, duration of the disease, number of IVIs before inclusion, treatment regimen and molecule injected. The patient, disease and treatment characteristics were recorded in a case report form, filled out by the retinal specialist investigator. The specific disease characteristics reported were the presence of IRF, SRF, atrophy, fibrosis, central retinal thickness (CRT) and the subtype of neovessel according to the recent consensus classification [16] (Supplementary File 1).

### 2.3. OCTA Acquisition

OCTA volumes were obtained using the swept-source OCTA PLEX Elite 9000 (Zeiss, Dublin, CA, USA). Each patient had a volumetric angiogram of 6 × 6 mm field of view centered on the fovea, comprised of 500 A-scans per B-scan and 500 B-scans per volume obtained at equally spaced positions. The angiography data were generated by processing two repeated OCT frames per B-scan position and using the eye tracking system (FastTrac, Carl Zeiss Meditec, Dublin, CA, USA). All acquisitions were performed by the same operator. Angiograms presenting motion or blinking artifacts were excluded. En-face angiograms were segmented automatically with the integrated PLEX Elite software, with the manual correction of segmentation errors, especially in cases of pigmented epithelium detachment (PED). Eyes with an OCTA signal strength below 8 (a measurement provided by the instrument software, ranging 0–10 and related to image quality), movement artifacts, or mask effects were not included in the study.

### 2.4. Image Analysis

**Figure 1:**
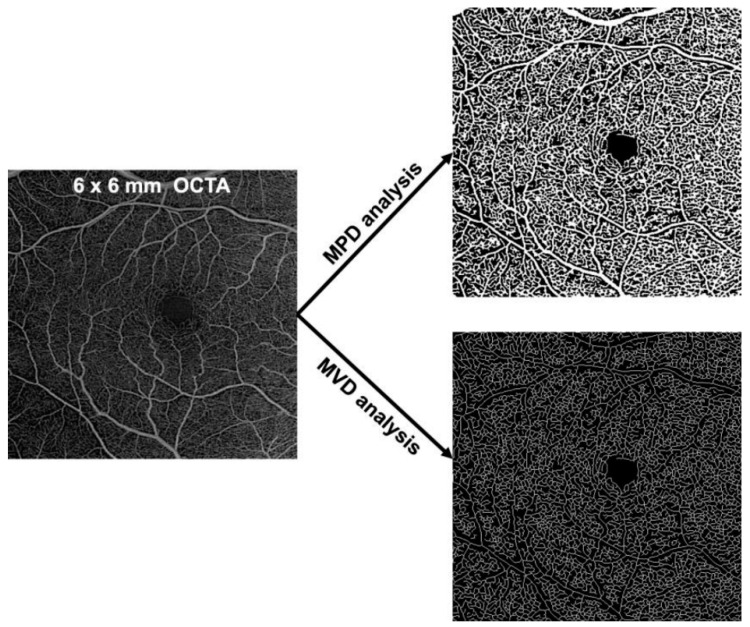
Post-acquisition analysis. MPD: mean perfusion density; MVD: mean vascular density.

Quantitative analysis of the retinal vascular density of the superficial capillary plexus (SCP) and the deep capillary plexus (DCP) was performed on the 6 × 6 mm OCTA for all patients. The SCP correspond to the capillary network in the ganglion cell layer and the DCP correspond to the capillary network between the outer boundary of the inner plexiform layer and the midpoint of the outer plexiform layer. OCTA volumes were anonymously exported by the software program after the careful verification and correction of possible segmentation errors. Exported OCTA volumes were uploaded to the ARI Network platform (https://arinetworkhub.com), a cloud-based collaborative and processing solution provided by the manufacturer. The vascular densities of the SCP and DCP were computed in the ARI Network platform using the Macular Density algorithm (version 0.7.1), a prototype algorithm provided by the instrument manufacturer (Carl Zeiss Meditec, Inc.) [17]. In short, this algorithm generates density and perfusion maps as follows: firstly, the SCP and DCP angiography images were generated using the segmentation algorithm as provided in the instrument and additional manual edits were made to correct any possible segmentation errors. Angiographic tail projections were corrected from the DCP image using a method equivalent to that available in the software program, removing projections from vessels appearing in the DCP image that were actually located within the SCP [18]. The resulting angiography images were resized to a canonical sampling density and processed using a proprietary algorithm to generate a binary mask indicating the presence of vasculature. This proprietary algorithm consists of the following steps: (1) histogram equalization; (2) hessian filtering (to enhance vessel patterns); (3) global thresholding using a computed “noise floor” (minimum signal level expected as image background) to binarize the image onto pixels belonging to vasculature or background; (4) morphological operations to clean up the resulting binary image from isolated pixels. After this binarization process, the ratio of segmented vessels (white pixels) over the total area gives the perfusion density values. The result is a number ranging from 0 (no perfusion) to 1 (fully perfused). The binarized image was further processed using a skeletonization algorithm (Figure 1). The ratio of the resulting skeleton (vessel length) over the total area constitutes the vessel density values, defined as the total length of perfused vasculature per unit area in a region of measurement. The result is a number with a minimum of 0 (no vessels) and an unbounded maximum. All methods were implemented in C++ and C# and complied into a dll. The workflow was managed using Matlab^®^ software (MathWorks, Natick, MA, USA).

### 2.5. Outcome Measures

The main outcome measure was the analysis of the SCP and DCP vascularization between groups. Retinal vascularization was assessed using the mean perfusion density (MPD) and mean vessel density (MVD) and computed for the whole 6 × 6 mm image. The secondary outcome measures were the analysis of variations in these values before and after exudation resolution (T1).

### 2.6. Statistical Analysis

As we expected more variability in the IRF ± SRF group due to the cystic areas, we planned to include twice as many patients in this group as in the SRF and control groups. The quantitative variables are presented as the mean and standard deviation (SD) and described as percentages for perfusion density data, and as continuous values for vascular density data. Factors potentially associated with MPD and MVD were studied using a univariable and multivariable linear regression analysis. Factors with *p* < 0.1 in the univariable analysis were entered into a multivariable model. The effect of time on the outcome variables was studied using a linear mixed model with age, fibrosis and atrophy as covariables. All analyses were performed using R (R Foundation for Statistical Computing, Vienna, Austria).

## 3. Results

Forty-six eyes (46 patients) were included in the present study. Two eyes were excluded due to poor image quality. A total of 44 eyes (44 patients) were analyzed: 22 eyes in 22 patients in the IRF ± SRF group, 11 eyes in 11 patients in the SRF group and 11 eyes in 11 patients in the healthy group. In the IRF ± SRF group, eight eyes were treatment-naive, and 14 eyes were already treated with anti-VEGF under a pro re nata (PRN) or treat-and-extend regimen. The eyes included in the IRF ± SRF group received a mean of 2.0 ± 1.0 IVIs between the first (T0) and the second (T1) OCTA imaging. The eyes included in the SRF group received a mean of 1.3 ± 0.5 IVIs between the two OCTA exams. Three patients in the SRF group were treatment-naive at the time of inclusion (Table 1). Before inclusion, the non-treatment-naive eyes received a mean of 9.1 ± 11.8 IVIs in the IRF ± SRF group and 18.6 ± 16.9 IVIs in the SRF group (*p* = 0.11).

### 3.1. Mean Perfusion Density (MPD) and Mean Vascular Density (MVD) at Baseline 

In the healthy group, MPD and MVD was 44.4 ± 2.2% and 19.7 ± 1.1%, respectively in the SCP and 34.1 ± 6.1 % and 16.2 ± 2.8 %, respectively in the DCP.

**Figure 2:**
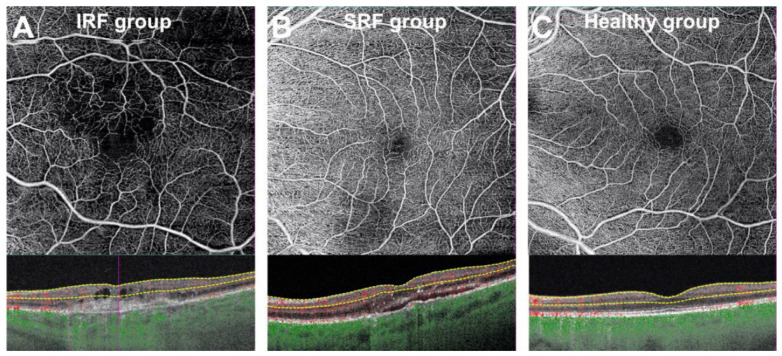
Optical coherence tomography angiography representative images of the superficial capillary plexus at T0 in eyes with intraretinal fluid (**A**) and subretinal fluid (**B**) compared to healthy subjects (**C**).

In the SCP at T0, eyes from the IRF ± SRF group showed significantly lower MPD and MVD than the healthy eyes (−7.0 95%CI (−9.6, −4.6), *p* < 0.001 and −3.1 95%CI (−4.3, −1.9), *p* < 0.001, respectively). Eyes from the SRF group showed no difference in MPD (*p* = 0.13) and MVD (*p* = 0.2) compared to the healthy eyes. These results remained significant for the IRF ± SRF group in the multivariable analysis (Table 2 and Table 3). Additionally, eyes from the IRF ± SRF group showed significantly lower MPD and MVD than the SRF group (*p* = 0.006 and *p* = 0.015, respectively) (Figure 2). Although lower MPD was found in cases of fibrosis (*p* = 0.045) and lower MVD was found in cases of fibrosis (*p* = 0.032) and atrophy (*p* = 0.048) in the SCP at T0, none of these factors were found to be significantly associated with these parameters in the multivariable analysis.

**Figure 3:**
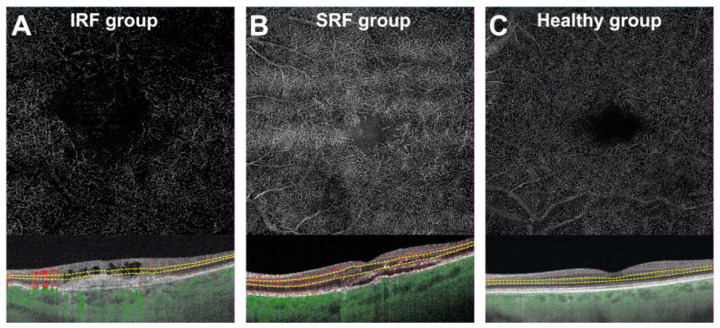
Optical coherence tomography angiography representative images of the deep capillary plexus at T0 in eyes with intraretinal fluid (**A**) and subretinal fluid (**B**) compared to healthy subjects (**C**).

In the DCP at T0, eyes from the IRF ± SRF group showed significantly lower MPD and MVD than the healthy eyes (−13.4 95%CI (−19.0, −8.0), *p* < 0.001 and –5.6 95%CI (−8.3, −3.0), *p* < 0.001, respectively). There was also a significant difference for eyes with SRF in comparison to healthy eyes in terms of MPD (−9.3 95%CI (−16.0, −3.1), *p* = 0.004) and MVD (−3.0 95%CI (−6.1, −0.05), *p* = 0.046). These results remained significant in the multivariable analysis except for MVD in the SRF group (*p* = 0.068) (Table 4 and Table 5). Furthermore, there were no differences between the IRF ± SRF and SRF groups in MPD and MVD (*p* = 0.3 and *p* = 0.11, respectively) (Figure 3). No other factors were found to be significantly associated with MPD or MVD variation in the univariable or the multivariable analysis.

### 3.2. Effect of Treatment/Time on Mean Perfusion Density (MPD) and Mean Vascular Density (MVD)

In the SCP, at T1 there was a significant increase in MPD (2.7 95%CI (0.2, 5.2), *p* = 0.048) and MVD (1.5 95%CI (0.5, 2.4), *p* = 0.008) for the IRF ± SRF group (Table 6 and Table 7). Although MPD and MVD increased after treatment up until the point of fluid resorption (T1), these values still remained lower than in the healthy eyes both for MPD (−4.3 95%CI (−7.8, −0.96), *p* = 0.013), and MVD (−1.6 95%CI (−2.9, −0.31), *p* = 0.017). Eyes with SRF did not show any significant changes in MPD (*p* = 0.36) or MVD (*p* = 0.33) at T1 in comparison to heathy eyes. Additionally, eyes from the IRF ± SRF group did not show any significant changes in MPD (*p* = 0.4) or MVD (*p* = 0.7) at T1 in comparison to eyes from the SRF group.

In the DCP, there was a significant increase in MPD (6.8 95%CI (2.1, 11.4), *p* = 0.009) at T1 in the IRF ± SRF group, but not in MVD (2.1 95%CI (−0.02, 4.34), *p* = 0.066). These retinal perfusion parameters at T1 still remained lower than in the healthy group for MPD (−6.6 95%CI (−12.0, −0.96), *p* = 0.021), and for MVD (−3.5 95%CI (−5.9, −1.1), *p* = 0.006). For eyes with SRF, no significant changes in MPD (*p* = 0.64) or MVD (*p* = 0.49) were found at T1. However, in eyes with SRF these values remained lower than in the healthy eyes for both MPD (−10.0 95%CI (−17.0, −3.9), *p* = 0.002), and MVD (−4.0 95%CI (−6.8, −1.2), *p* = 0.006). Additionally, eyes from the IRF ± SRF group did not show any significant changes in MPD (*p* = 0.2) or MVD (*p* = 0.7) at T1 in comparison to eyes from the SRF group.

## 4. Discussion

In the present study, we analyzed the retinal vascular changes visualized on OCTA in exudative AMD according to the location of the fluid. We showed that patients with IRF ± SRF have lower MPD in both the SCP and the DCP in comparison to healthy individuals, and MPD was only lower in the DCP in eyes with SRF. After exudation resorption, eyes with IRF showed a significant increase in MPD in both plexuses, in comparison to eyes with SRF in which MPD remained constant. However, at the time of fluid resorption (T1), eyes with previous IRF ± SRF still had lower MPD than healthy eyes in both the SCP and DCP, whereas eyes with SRF only had lower MPD in the DCP. In this study, the same results were found for MVD. However, unlike for MPD, all vessels are treated equally. Regarding MPD, larger vessels influence the measurement more than smaller capillaries and can therefore hide the loss of individual capillaries. MVD is more sensitive to the loss of individual capillaries as it measures all of the retinal plexus vasculature equally. The downside of this method is its lower signal to noise ratio.

Several studies have reported abnormal vascular changes in exudative or atrophic AMD [19,20]. This is not surprising as high blood pressure, smoking and obesity are risk factors of the disease [21]. Some authors found arteriolar narrowing and arteriovenous crossing in patients suffering from AMD and suggested a possible association with disease progression [19,22]. Toto et al. found a decrease in perfusion density in the SCP in patients with a high risk of developing late stage atrophic AMD [23]. Although the presence of ischemic areas in the choriocapillaris is a well-known feature of atrophic AMD [24], some studies have also found reduced retinal vessel density in this form of the disease [25,26]. For exudative AMD, same observation has been made for choriocapillaris [27], however, the present study is the first to describe an alteration in retinal perfusion. Surprisingly, there is a significant increase in MPD in eyes with IRF after exudation resorption, meaning that a reperfusion of the cystic area does occur, in contrast to vaso-occlusive diseases. Indeed, in DME and RVO, it has been shown that MPD does not increase after IRF resolution [9,10].

The physiopathology of IRF in exudative AMD is unclear [28]. Gass hypothesized that the extension of choroidal neovessels beneath the retinal capillary-free zone might disrupt the photoreceptors–outer retinal membrane complex. This might lead to the intraretinal migration of subretinal exudate if there are not enough retinal capillaries in this area to remove it [29]. It has been shown that AMD patients with IRF experience worse visual outcomes than patients with SRF alone [30]. In the present study, although MPD and MVD increased between T0 and T1 in eyes with IRF, they remained lower than in the healthy eyes. This could explain the worse visual outcomes associated with IRF. In contrast, eyes with SRF only had significantly lower MPD and MVD in the DCP and there was no significant increase at the time in fluid resorption (T1). This might be explained by the longer duration of disease found for eyes with SRF in the present cohort, which could potentially lead to chronic alterations to the retinal vascular plexus. It should be noted that fluid exhibits some layer-dependent properties in exudative AMD. IRF normally appears above the outer plexiform layer, thus involving DCP and sometimes SCP. In contrast, SRF is accumulated beneath the outer segment layer and do not affect both retinal capillary plexus [31].

Taken together, our analysis of OCTA images showed only a partial reperfusion and vascular reorganization of cystic areas after exudation resorption in eyes with IRF. Knowing that patients with IRF experienced worse visual outcomes than patients with SRF [32,33], our results suggest that patients presenting IRF secondary to exudative AMD should be treated rapidly, as the reperfusion capacity of the cystic areas appears to be overstretched and areas of non-perfusion persist despite edema resolution.

Some studies have reported an association between anti-VEGF treatment and retinal ischemic phenomenon, and hypothesized a possible arteriolar vasoconstriction inducing a decrease in blood flow after injection [34,35,36]. Recently, Mastropasqua et al. have observed a significant decrease in macular vascular density on OCTA one month after anti-VEGF injection in patients with exudative AMD without IRF [37]. In the present study, the secondary analysis comparing treatment naïve and non-naïve patients did not find any association between anti-VEGF treatment and MPD or MVD. Although no conclusion can be made at this stage, further studies are needed to evaluate if the vascular alterations occur in the early stages of the disease, before the first treatment injections. This hypothesis argues for fast and intensive pro-active treatment, but this assumption needs to be clinically verified. Finally, no differences were found between neovascular subtypes in terms of retinal plexus reperfusion.

The main limitation of the present study was the small number of eyes included in the different fluid groups. Despite matching the control patients for age and duration of disease, several other factors including atrophy, fibrosis or treatment regimen were also present. Moreover, the time from the last injection to OCTA imaging was not the same for all patients and could have modified the capillary alterations observed in this study. In order to limit these biases, we used a univariable and multivariable linear regression analysis to take into account potential confounding factors. However, due to the lack of data, we did not integrate ocular axial length or refractive error into this model, knowing that they can change retinal vessel density measurement [38]. In this case, MPD and MVD values might be changed by an optical magnifying effect caused by the edema itself. Further studies are needed to analyze the impact of such an optical effect on MPD and MVD measurements. Another limitation was the management of segmentation artifacts on OCTA in cases where IRF disorganizes the retinal architecture [39]. For instance, some studies excluded patients with IRF, particularly when analyzing choriocapillaris vasculature [7,39,40]. Pigmented epithelium detachment could also have disorganized retinal segmentation on OCTA in the IRF and SRF groups and could thereby have modified the retinal vascular analyses. However, all OCTA images and segmentation were systematically reviewed, and any segmentation errors were modified manually before imaging analysis.

## 5. Conclusions

The present study shows that retinal vascular changes occur in patients with IRF in cases of AMD. However, cystic areas can be partially re-perfused after edema resolution. These results could partially explain the worse visual outcomes in eyes presenting IRF induced by macular neovascularization. Further large-scale studies are needed to confirm our hypothesis.

## Figures and Tables

**Table 1:** Patient characteristics.

	IRF ± SRF Group (*n* = 22)	SRF Group (*n* = 11)	Healthy Group (*n* = 11)
Mean age, years (SD)	81.7 (8.3)	79.9 (5.8)	78.6 (2.8)
Sex (male/female), *n*	5/17	5/6	6/5
Laterality (R/L), *n*	10/12	7/4	5/6
Mean disease duration, months (SD)	22.6 (26.1)	33.7 (29.3)	-
Mean duration between T0 and T1, months (SD)	2.7 (1.7)	1.2 (0.4)	-
Mean number of IVI before inclusion, *n* (SD)	9.1 (11.8)	18.6 (16.9)	-
Mean number of IVI between T0 and T1, *n* (SD)	2.0 (1.0)	1.3 (0.5)	-
Treatment regimen, *n* (%) Pro re nata Treat and Extend Treatment-naive	7 (31.8) 7 (31.8) 8 (36.4)	1 (9.1) 7 (63.6) 3 (27.3)	-
Presence of SRF, *n* (%)	6 (27.3)	11 (100)	-
Presence of IRF, *n* (%)	22 (100)	0 (0)	-
Presence of atrophy, *n* (%)	13 (59.1)	2 (18.2)	-
Presence of fibrosis, *n* (%)	8 (36.4)	1 (9.0)	-
Mean BCVA at T0, ETDRS (SD)	56.8 (19.7)	72.0 (15.5)	-
Mean BCVA at T1, ETDRS (SD)	58.1 (19.8)	72.7 (16.0)	-
Type of anti-VEGF, *n* (%) Aflibercept Ranibizumab	19 (86.4) 3 (13.6)	11 (100) 0 (0)	-

BCVA: best-corrected visual acuity; ETDRS: early treatment diabetic retinopathy study; IRF: intraretinal fluid; IVI: intravitreal injection; R/L: right/left; SD: standard deviation; SRF: subretinal fluid; VEGF: vascular endothelial growth factor.

**Table 2:** Mean perfusion density (MPD) in the superficial capillary plexus (SCP) according to different factors in the univariate and multivariate analyses.

	MPD SCP			
	Univariable		Multivariable	
	Estimate (95%CI)	*p*-Value	Estimate (95%CI)	*p*-Value
Fluid group				
Healthy	Ref		Ref	
IRF ± SRF	−7.0 (−9.6, −4.6)	<0.001	−5.7 (−8.7, −2.7)	<0.001
SRF	−2.2 (−5.1, 0.7)	0.13	−1.8 (−4.7, 1.1)	0.2
Age	−0.2 (−0.4, 0.01)	0.061	−0.1 (−0.3, 0.05)	0.2
Sex (Male)	2.1 (−0.7, 4.9)	0.14	-	-
Disease duration	−0.02 (−0.08, 0.04)	0.5	-	-
Number of IVI	0.02 (−0.09, 0.1)	0.7	-	-
Atrophy	−2.8 (−5.7, 0.1)	0.062	−0.7 (−3.4, 1.9)	0.6
Fibrosis	−3.3 (−6.5, −0.07)	0.045	−1.7 (−4.6, 1.2)	0.2
MNV subtype				
Type 1	Ref			
Type 2	−1.5 (−6.3, 3.4)	0.5	-	-
Type 3	1.3 (−2.1, 4.7)	0.4	-	-
Anti-VEGF				
Aflibercept	Ref			
Lucentis	0.4 (−5.0, 5.7)	0.9	-	-
Treatment				
Naive	Ref			
PRN	−0.4 (−4.6, 3.7)	0.8	-	-
TAE	0.8 (−2.8, 4.4)	0.6	-	-

IRF: intraretinal fluid; IVI: intravitreal injection; MNV: macular neovessel; PRN: Pro re nata; SCP: superficial capillary plexus; SRF: subretinal fluid; TAE: treat and extend; VEGF: vascular endothelial growth factor.

**Table 3:** Mean vascular density (MVD) in the superficial capillary plexus (SCP) according to different factors in the univariate and multivariate analyses.

	MVD SCP			
	Univariable		Multivariable	
	Estimate (95%CI)	*p*-Value	Estimate (95%CI)	*p*-Value
Fluid group				
Healthy	Ref		Ref	
IRF ± SRF	−3.1 (−4.3, −1.9)	<0.001	−2.4 (−3.8, −0.9)	0.002
SRF	−0.9 (−2.3, 0.5)	0.2	−0.7 (−2.1, 0.7)	0.3
Age	−0.07 (−0.2, 0.02)	0.14	−0.03 (−0.1, 0.04)	0.4
Sex (Male)	1.0 (−0.3, 2.3)	0.14	-	-
Disease duration	−0.01 (−0.03, 0.02)	0.6	-	-
Number of IVI	0.01 (−0.04, 0.07)	0.6	-	-
Atrophy	−1.4 (−2.8, −0.01)	0.048	−0.4 (−1.7, 0.9)	0.5
Fibrosis	−1.7 (−3.2, −0.1)	0.032	−0.9 (−2.3, 0.5)	0.2
MNV subtype				
Type 1	Ref			
Type 2	−0.9 (−3.2, 1.3)	0.4	-	-
Type 3	0.9 (−0.7, 2.5)	0.3	-	-
Anti-VEGF				
Aflibercept	Ref			
Lucentis	0.4 (−2.2, 2.9)	0.8	-	-
Treatment				
Naive	Ref			
PRN	−0.07 (−2.0, 1.9)	>0.9	-	-
TAE	0.8 (−1.3, 2.1)	0.6	-	-

IRF: intraretinal fluid; IVI: intravitreal injection; MNV: macular neovessel; PRN: Pro re nata; SCP: superficial capillary plexus; SRF: subretinal fluid; TAE: treat and extend; VEGF: vascular endothelial growth factor.

**Table 4:** Mean perfusion density (MPD) in the deep capillary plexus (DCP) according to different factors in the univariate and multivariate analyses.

	MPD DCP			
	Univariable		Multivariable	
	Estimate (95%CI)	*p*-Value	Estimate (95%CI)	*p*-Value
Fluid group				
Healthy	Ref		Ref	
IRF ± SRF	−13.4 (−19.0, −8.0)	<0.001	−13.0 (−19.0, −5.9)	<0.001
SRF	−9.3 (−16.0, −3.1)	0.004	−9.2 (−16.0, −2.7)	0.007
Age	−0.02 (−0.4, 0.4)	>0.9	0.1 (−0.2, 0.5)	0.4
Sex (Male)	4.1 (−1.5, 9.7)	0.15	-	-
Disease duration	−0.06 (−0.2, 0.04)	0.2	-	-
Number of IVI	−0.10 (−0.3, 0.1)	0.3	-	-
Atrophy	−2.9 (−8.3, 2.6)	0.3	−1.1 (−7.1, 4.9)	0.7
Fibrosis	−2.9 (−9.0, 3.2)	0.3	−1.5 (−8.0, 5.0)	0.6
MNV subtype				
Type 1	Ref			
Type 2	−4.4 (−13.0, 4.3)	0.3	-	-
Type 3	2.4 (−3.6, 8.3)	0.4	-	-
Anti-VEGF				
Aflibercept	Ref			
Lucentis	−1.9 (−12.0, 7.7)	0.7	-	-
Treatment				
Naive	Ref			
PRN	−2.8 (−10.0, 4.6)	0.4	-	-
TAE	−1.0 (−7.4, 5.5)	0.8	-	-

DCP: deep capillary plexus; IRF: intraretinal fluid; IVI: intravitreal injection; MNV: macular neovessel; PRN: Pro re nata; SRF: subretinal fluid; TAE: treat and extend; VEGF: vascular endothelial growth factor.

**Table 5:** Mean vascular density (MVD) in the deep capillary plexus (DCP) according to different factors in the univariate and multivariate analyses.

	MVD DCP			
	Univariable		Multivariable	
	Estimate (95%CI)	*p*-Value	Estimate (95%CI)	*p*-Value
Fluid group				
Healthy	Ref		Ref	
IRF ± SRF	−5.6 (−8.3, −3.0)	<0.001	−5.3 (−8.6, −2.1)	0.002
SRF	−3.0 (−6.1, −0.05)	0.046	−2.9 (−6.1, 0.2)	0.068
Age	−0.09 (−0.3, 0.1)	0.3	−0.02 (−0.2, 0.1)	0.8
Sex (Male)	1.9 (−0.7, 4.1)	0.15	-	-
Disease duration	−0.02 (−0.07, 0.03)	0.4	-	-
Number of IVI	−0.01 (−0.1, 0.09)	0.8	-	-
Atrophy	−1.3 (−4.1, 1.4)	0.3	−0.5 (−3.4, 2.4)	0.7
Fibrosis	−0.8 (−3.9, 2.2)	0.6	−0.1 (−3.0, 3.3)	>0.9
MNV subtype				
Type 1	Ref			
Type 2	−0.3 (−4.7, 4.2)	0.9	-	-
Type 3	0.4 (−2.6, 3.5)	0.8	-	-
Anti-VEGF				
Aflibercept	Ref			
Lucentis	−2.0 (−6.7, 2.8)	0.4	-	-
Treatment				
Naive	Ref			
PRN	−1.2 (−4.8, 2.4)	0.5	-	-
TAE	−1.1 (−2.1, 4.3)	0.5	-	-

DCP: deep capillary plexus; IRF: intraretinal fluid; IVI: intravitreal injection; MNV: macular neovessel; PRN: Pro re nata; SRF: subretinal fluid; TAE: treat and extend; VEGF: vascular endothelial growth factor.

**Table 6:** Effect of time on mean perfusion density (MPD) according to the patient group and plexus analyzed. The analyses were performed using a multivariable linear mixed effects model including age, fibrosis and atrophy as covariates. The estimate gives the increase or decrease in the variable between T0 and T1 independently of age, fibrosis and atrophy.

	T0	T1	Estimate (95%CI)	*p*-Value
Healthy group SCP DCP	44.4 ± 2.2 34.1 ± 6.1		-	-
IRF **±** SRF group SCP DCP	37.4 ± 4.1 20.7 ± 7.8	40.1 ± 5.8 27.5 ± 8.4	2.7 (0.2, 5.2) 6.8 (2.1, 11.4)	0.048 0.009
SRF group SCP DCP	42.2 ± 2.3 24.8 ± 6.9	41.4 ± 3.0 23.8 ± 6.3	−0.8 (−2.6, 0.9) −1.0 (−5.7, 3.8)	0.36 0.64

DCP: deep capillary plexus; IRF: intraretinal fluid; SCP: superficial capillary plexus; SRF: subretinal fluid.

**Table 7:** Effect of time on mean vascular density (MVD) according to the patient group and plexus analyzed. The analyses were performed using a multivariable linear mixed effects model including age, fibrosis and atrophy as covariates. The estimate gives the increase or decrease in the variable between T0 and T1 independently of age, fibrosis and atrophy.

	T0	T1	Estimate (95%CI)	*p*-Value
Healthy group SCP DCP	19.7 ± 1.1 16.2 ± 2.8		-	-
IRF **±** SRF group SCP DCP	16.6 ± 1.9 10.6 ± 3.7	18.1 ± 2.1 12.7 ± 3.6	1.5 (0.5, 2.4) 2.1 (−0.02, 4.34)	0.008 0.066
SRF group SCP DCP	18.8 ± 1.3 13.2 ± 2.9	18.3 ± 1.5 12.2 ± 2.7	−0.5 (−1.2, 0.4) −1.0 (−3.4, 1.6)	0.33 0.49

DCP: deep capillary plexus; IRF: intraretinal fluid; SCP: superficial capillary plexus; SRF: subretinal fluid.

## Data Availability

All data are available upon request to the corresponding author.

## Supplementary Materials

The following are available online at https://www.mdpi.com/article/10.3390/jcm10071524/s1, File 1, case report form for each anonymized patient. BCVA: best-corrected visual acuity; CNV: choroidal neovessel; CRT: central retinal thickness; DCP: deep capillary plexus; F: female; IRF: intraretinal fluid; IVT: intravitreal injection; L: left; M: male; MPD: mean perfusion density; MVD: mean vascular density; PRN: pro renata; R; right; SCP: superficial capillary plexus; SRF: subretinal fluid; TAE: treat and extend.
