# Supplementary material for: Retinal Vascularization Analysis on Optical Coherence Tomography Angiography before and after Intraretinal or Subretinal Fluid Resorption in Exudative Age-Related Macular Degeneration: A Pilot Study
Source: J Clin Med. 2021 Apr 6;10(7):1524. doi: 10.3390/jcm10071524 (PMC8038669; doi:10.3390/jcm10071524)
Supplement: Supplementary file 1 [file jcm-10-01524-s001.pdf]

IDENT	GROUP	AGE	SEX	LAT	DURATION	CNV subtype	NB IVT T0	NB IVT T1-T0	T1-T0 (days)
1	1	97	F	L	84	1	0	1	63
2	1	83	M	L	0	1	0	2	56
3	1	81	F	L	19	1	12	2	189
4	1	88	F	R	84	1	44	3	28
5	1	62	F	R	36	2	4	2	63
6	1	85	F	R	0	3	0	3	136
7	1	85	M	L	34	1	14	1	35
8	1	77	F	L	33	2	11	1	84
9	1	72	F	R	25	2	10	3	175
10	1	96	F	L	18	1	6	2	58
11	1	85	M	R	0	1	0	2	70
12	1	81	F	L	0	3	0	2	102
13	1	87	F	R	0	3	0	1	33
14	1	83	F	R	57	1	29	1	35
15	1	81	F	L	6	3	7	2	181
16	1	65	M	L	2	3	2	2	69
17	1	77	F	R	0	2	0	1	30
18	1	81	F	L	30	1	28	3	119
19	1	81	M	L	35	3	13	3	168
20	1	89	F	R	0	3	0	3	175
21	1	77	F	L	35	3	12	1	35
22	1	84	F	R	0	1	0	1	28
23	2	82	F	R	17	1	12	1	28
24	2	75	F	L	49	3	14	1	63
25	2	83	M	R	0	3	0	2	56
26	2	88	M	R	0	1	0	2	45
27	2	75	M	R	0	1	0	2	365
28	2	87	F	L	39	3	16	1	35
29	2	72	F	R	39	1	35	1	49
30	2	74	F	L	18	1	9	1	42
31	2	75	M	R	53	1	45	1	28
32	2	85	M	L	72	1	35	1	42
33	2	83	F	R	84	1	39	1	42
34	3	79	F	L					
35	3	80	F	R					
36	3	76	F	L					
37	3	76	F	R					
38	3	76	F	L					
39	3	74	M	R					
40	3	79	M	R					
41	3	79	M	L					
42	3	82	M	L					
43	3	82	M	R					
44	3	82	M	L					

BCVA T0	BCVA T1	CRT T0	CRT T1	Atrophy	Fibrosis	SRF	MPD SCP T0	MPD SCP T1	MPD DCP T0
NC	NC	343	227	0	1	0	30,97	19,79	15,86
65	75	362	270	0	0	0	28,93	40,41	7,4
26	30	833	446	1	1	0	38,91	40,01	22,69
62	68	246	209	1	0	0	29,22	41,91	5,79
57	49	349	255	1	1	0	39,91	38,23	18,17
72	71	275	220	1	0	1	35,53	38,38	15,2
15	17	442	321	1	1	0	37,99	47,13	23,61
23	27	388	347	1	1	0	36,88	39,51	17,05
47	47	284	256	1	1	0	33,15	46,39	14,57
72	65	371	269	1	1	0	39,6	37,28	28,22
62	56	367	293	1	0	1	36,5	44,5	19,07
35	45	557	272	0	0	1	41,59	40,79	15,72
58	63	423	220	0	0	0	37,72	42,57	27,79
33	30	444	307	1	1	0	33,65	30,37	15,99
55	54	314	232	1	0	1	41,82	45,45	28,99
78	78	289	261	0	0	0	41,9	43,17	28,88
71	75	436	296	0	0	1	39,13	43,4	20,13
81	87	242	222	1	0	0	39,43	38,68	25,95
61	57	372	279	0	0	0	40,33	44,22	18,34
82	78	268	219	0	0	0	40,81	41,02	34,83
65	65	276	188	1	0	0	35,11	38,32	16,94
73	83	353	227	0	0	1	43,25	40,23	35,69
46	55	262	190	0	0		38,82	35,89	26,35
84	87	272	266	0	0		46,05	44,21	37,13
70	75	306	267	0	1		38,28	40,42	23,81
70	62	317	273	1	0		42,64	39,3	29,35
88	87	261	243	1	0		41,97	37,17	26,21
73	75	NC	310	0	0		41,41	45,4	18,51
88	90	336	399	0	0		41,27	43,02	15,17
NC	NC	268	238	0	0		42,97	41,48	18,41
46	41	268	258	0	0		42,75	44,23	19,97
71	70	378	266	0	0		45,17	42,62	34,81
84	85	303	310	0	0		43,3	41,9	22,66
							45,47		40,58
							44,65		35,68
							44,44		31,29
							39,48		19,11
							44,29		36,66
							45,75		36,53
							43,93		28,45
							45,6		35,52
							47,98		37,31
							45,49		40,44
							41,79		33,86

MPD DCP T1	MVD SCP T0	MVD SCP T1	MVD DCP T0	MVD DCP T1	MOLECULE	REGIMEN
7,72	13,52	12,65	7,76	6,77	EYLEA	PRN
23,6	12,81	18,02	3,73	11,73	EYLEA	NAIF
17,48	17,09	17,49	10,93	8,16	EYLEA	PRN
32,52	13,39	17,81	3,07	15,63	EYLEA	TAE
24,59	16,66	16,94	9,17	12,18	EYLEA	PRN
26,22	15,49	19,6	7,56	12,88	EYLEA	NAIF
35,1	17,01	20,27	11,69	16,26	EYLEA	PRN
36,5	16,13	16,86	8,44	12,81	LUCENTIS	TAE
34,73	14,66	20,92	16,42	7,34	EYLEA	TAE
23,75	18,44	17,47	13,58	11,58	LUCENTIS	TAE
30,47	15,66	19,43	9,48	14,41	EYLEA	NAIF
17,42	18,45	18,42	6,87	8,56	LUCENTIS	NAIF
31,97	16,93	19,26	13,79	15,91	EYLEA	NAIF
12,19	14,97	13,31	7,85	5,98	EYLEA	TAE
41,26	19,07	20,81	14,32	19,33	EYLEA	PRN
32,02	18,77	18,95	13,77	15,18	EYLEA	TAE
35,98	17,41	19,33	10,12	16,64	EYLEA	NAIF
24,39	16,78	16,62	12,69	12,01	EYLEA	TAE
36,34	18,65	19,88	9,35	17,69	EYLEA	PRN
28,71	18,52	18,13	16,85	13,71	EYLEA	NAIF
28,33	15,69	16,78	8,45	13,72	EYLEA	PRN
24,08	19,4	18,34	16,79	11,71	EYLEA	NAIF
23,68	16,73	15,82	12,69	11,54	EYLEA	TAE
33,42	21,11	20,23	18,16	16,32	EYLEA	TAE
27,35	16,62	18,07	11,6	13,46	EYLEA	NAIF
18,2	19,04	17,5	14,18	8,88	EYLEA	NAIF
24,1	18,61	16,53	12,98	11,56	EYLEA	NAIF
33,93	19,18	20,99	8,83	15,58	EYLEA	PRN
17,78	19,15	19,57	7,75	9,41	EYLEA	TAE
14,83	18,04	17,43	13,73	8,11	EYLEA	TAE
17,7	19,39	19,1	16,58	13,65	EYLEA	TAE
23,79	20,21	18,24	16,7	11,49	EYLEA	TAE
27,13	18,48	18,42	11,68	14,71	EYLEA	TAE
	19,98		18,83			
	19,72		16,89			
	20,14		15,13			
	17,15		9,12			
	19,7		17,25			
	20,67		17,75			
	19,15		13,65			
	19,84		16,99			
	21,45		17,84			
	18,74		16,19			
	19,91		18,85			
